# Diagnostic Value of Non Stress Test in Latent Phase of Labor and Maternal and Fetal Outcomes

**DOI:** 10.5539/gjhs.v7n2p177

**Published:** 2014-10-28

**Authors:** Shiva Raouf, Fatemeh Sheikhan, Shirin Hassanpour, Soheila Bani, Rogayye Torabi, Neda Shamsalizadeh

**Affiliations:** 1 Nursing & Midwifery Faculty of Tabriz University of Medical Sciences, Tabriz, Iran; 2 Department of midwifery, Khalkhal Branch, Islamic Azad University, Khalkhal, Iran; 3 Midwifery Group Educator of Nursing and Midwifery Faculty of Tabriz University of Medical Sciences, Tabriz, Iran; 4 Bachelor Science of Midwifery, Nursing and Midwifery Department, Tabriz University of Medical Sciences, Tabriz, Iran; 5 Decker School of Nursing (DSON), Binghamton University (SUNY), New York, USA

**Keywords:** neonatal outcomes, maternal outcomes, pregnant women, non-reactive NST, Reactive NST, non stress test

## Abstract

**Purpose:**

The Non stress test (NST) is one of the significant diagnostic fetal wellbeing tests. The purpose of this study is to assess diagnostic value of NST during latent phase of labor by considering maternal and neonatal outcomes.

**Subjects:**

This case control study was performed on 450 healthy pregnant women with gestational ages between 38-42 weeks in AL-Zahra teaching hospital in Tabriz, Iran. All participants underwent NST after being admitted to labor during their latent phase of delivery. Participants were divided into two groups including the study group which included 150 participants with non-reactive NST results whereas 300 subjects with reactive NST results assigned in the control group. Subjects in both groups were hospitalized for pregnancy termination because of the delivery time. In order to find out the importance of routine performance of NST during delivery, the relationship between NST results and maternal and fetal outcomes was evaluated. Several criteria including type of delivery, meconium defecation, descent arrest, bradycardia, Apgar score, and still birth were compared between two groups.

**Results:**

Findings of this study showed that descent arrest occurred in 2.7% of the subjects in the study group, whereas it occurred in 4.7% of the participants in the control group (*p*=0.44). Bradycardia found in 28% of the participants in study group and 3.3% of the control group (*p*<0.001). The low Apgar score was found in 2.7% of case group however; no the low Apgar score detected in the control group. Meconium defecation observed in 11.3% of the subjects in the study group and 9.7% of the participants in control group (*p*=0.62). The amount of stillbirth was 2.7% in the study group and no stillbirths were found in control group. There was a significant difference between the results of both groups in terms of bradycardia, low Apgar score and cesarean section.

**Conclusion:**

Results of this study revealed that participants in study group with nonreactive NST results had more fetal complications than those with reactive NST results. NST was found to be a valuable diagnostic test for diagnosis of fetal distress during delivery in the latent phase. These findings of this study suggest that NST should be performed routinely as a valuable diagnostic test during the latent phase of delivery.

## 1. Introduction

The maternal mortality rate has significantly reduced in developing countries. Thus, the focus has shifted toward fetal health. The fetus is a second patient with a high risk of morbidity and mortality. Gestational ages between 37 and 42 weeks are defined as a term pregnancy. By using diagnostic tests, 56% of the stillbirths can be preventable (Zuspan & Zuspan, 1994; Jams, Steer, Weiner, & Gonic, 2000). Non Stress test (NST) is one of the antepartum surveillance techniques that is used to evaluate the fetal wellbeing and to rule out fetal distress (Hassanzadeh, 2004). The basis of NST is the increase of fetal heart rate in response to fetal movements. The rise of at least 15 bpm lasting for 15 seconds or more, during a period of 20 minutes is the definition of fetal heart rate increase (FHR). A sufficient number of fetal movements is one of the indicators of a healthy fetus (Christopher, Harman, & Frank, 2000; Leng, & Duff, 2001; Hasanpour et al., 2013). Normally, fetal movements lead to an increase in FHR (Menihan & Kopel, 2007) and are directly related to the sympathetic and parasympathetic autonomic nervous systems which do not exist before 26-27 weeks (Zuspan & Zuspan, 1994; Jams et al., 2000). When FHR increases in response to fetal movements, the fetus is considered healthy (Gabbe, Niebyl, & Simposn, 2000; Gilbert, & Hamon, 2003). NST result is one of the determinant factors for health providers to decide between waiting, performing further assessment or starting labor induction. Although NST is known as a valuable diagnostic test and is used as a diagnostic test during third trimester of the pregnancy, currently it is not performed routinely during labor. At that time both mother and the fetus need regular assessments based on the different stages and their risk statuses. Although the midwife or the health care provider has to take care of both mother and the baby, the fetus cannot be observed directly. General guidelines for fetal assessments during labor include FHR and amniotic fluid assessments. Maternal assessments also are related to the fetal wellbeing. General maternal assessments are vital signs, contractions, labor progression (using vaginal examination), amount of intake and output and the mother’s response to labor. Although FHR response to fetal movement is one of criteria of healthy fetus, assessment of FHR is not performed routinely during labor. Labor includes three stages including: stage one or full cervical dilatation, stage two or infant delivery, and stage three or passage of the placenta. Stage one consists of three phases of latent, active, and deceleration. The latent phase is the initial phase of the labor during which contractions become regular and cervical dilatation reaches 3–4 cm. Many pregnant women arrive at hospitals during their first stage of labor. Midwives and nurse midwives provide a fundamental role during labor while providing support during delivery processes, developing a meaningful experience, rapid detection of the possible complications, and prevention of mortality and morbidity. NST is one of the surveillance techniques that can avoid unnecessary interventions in childbirth and associated complications for both mother and fetus. Guidelines for NST, ultrasound examination and Doppler examinations are based on limited evidence (Tveit, Saastad, Stray-Pedersen, Børdahl, Flenady, Fretts, & Frøen, 2009; Olesen, & Svare, 2004; Frøen, Tveit, Saastad, Børdahl, Stray-Pedersen, & Heazell, 2008). Therefore, in order to show the diagnostic value of NST during the latent phase of the labor and its role in diagnosis of fetal complications, this study performed to test the association between NST results and maternal and neonatal outcomes in term pregnant women referred to Alzahra hospital of Tabriz in 2013.

## 2. Materials and Methods

This descriptive study was performed from April to November 2013, in Al Zahra educational you said teaching before hospital affiliated with Tabriz University of Medical Sciences. Data gathering tools included: A personal and social demographic questionnaire, an obstetric checklist, and forms for NST reports. Information about the study was given to the eligible participants and a written informed consent was obtained from each of them. The study population included 450 pregnant women including 150 subjects in case group and 350 participants in control group. The gestational ages of all of the subjects were between 38-42 weeks. All participants had been referred to emergency department of Al Zahra educational hospital for termination of pregnancy due to natural delivery timing. All participants underwent NST after being admitted to the labor department during their latent phase of delivery. By considering ∝=5% and the power=80% and the Apgar score difference of 2 between two groups, 150 subjects were estimated for each group. In order to increase the study accuracy, the control group participants were doubled in size in comparison to the case group (300 subjects).

For this study, the following inclusion criteria for study group included; subjects who were willing to participate in the study, singleton pregnancies, gestational ages between 38-42 weeks, normal FHR between 120 and 160 beats per minute, having no known systemic disease during or before pregnancy, not having pelvic diseases, non- smoking and no alcohol consumption, having no history of previous Cesarean section, infant congenital anomaly or stillbirth and having non-reactive results in NST (less than 2 increase in fetal heart rate during 20 min) and being hospitalized because of natural delivery timing. Inclusion criteria for the control group only differed from the study group in terms of reactive NST results.

The tools for data collection included a checklist which was devised based on the available information used in previous research including: maternal demographic characteristics and midwifery specifications, NST results, and maternal and fetal outcomes. The mothers’ personal and midwifery specifications in the checklist consisted of age, education, income level, number of deceased and alive children, history of previous abortion, the first date of last menstruation period (LMP), gestational age based on LMP, first trimester sonography, and NST reports.

Maternal and fetal outcomes which were evaluated in this study included: the Apgar score, neonatal hospitalization status, duration of hospitalization in NICU, type of childbirth (Natural vaginal delivery or Cesarean section), cause of Cesarean section (bradycardia, descent arrest, and/or meconium defecation).

The FHR monitoring device used in this research was manufactured by Japanese Toyota Company, model MT325, which is one of the most prestigious companies producing medical equipment and as a result, has scientific and practical validity and reliability. NST results and maternal and fetal outcomes were compared between the study and control groups to determine the diagnostic value of the NST during latent phase of delivery. Data were analyzed by SPSS 17 using Chi square test or Fisher’s exact test, Spearman’s correlation coefficient, and Mann – Whitney U test. A logistic regression model was used in order to evaluate the predictor variables with *p*-value equal to or less than 0.05 considered as statistically significant.

## 3. Results

In this study, the mean age for pregnant mothers was 26.47±5.55 years in the case group and 25.56±5.22 years in the control group (*p*=0.09). The mean gestational age was 39.06±0.86 weeks in the case group and 38.90±1.14 weeks in the control group (*p*=0.093). In terms of the job, all participants were housewives. There was no statistically significant difference between the two groups in terms of individual, social and midwifery specifications.

From 150 participants in study group, 17 cases of meconium defecation were reported (11.3%) and 15 cases led to Cesarean section. Out of 15 cesarean section deliveries, 4 infants were hospitalized in NICU. From 300 mothers in control group, there were 29 cases of meconium defecation (9.7%). 24 out of 29 cases with meconium defecation underwent Cesarean section (*p*=0.62) from which 2 infants were transferred to the NICU. There was no statistically significant difference between the two groups for meconium defecation. There was no placenta abruption in the study group however 2 placenta abruption occurred in the control group (0.7%) and both infants were treated in the NICU. There was no statistically significant difference between the two groups for placenta abruption. There were 4 cases with descent arrest in the study group (2.7%) each of which led to Cesarean section. There were 14 cases of descent arrest reported in the control group (4.7%) which ended with Cesarean section (*p*=0.44).

Fetal bradycardia occurred in 42 subjects in the study group (28%) which led to Cesarean section in all of them and from which 19 infants were hospitalized in NICU. Fetal bradycardia reported in 10 participants in the control group (3.3%) which ended with Cesarean section in all of them and one infant was hospitalized in NICU. There was a statistically significant difference for fetal bradycardia between two groups (*p*<0.001).

In the study group, 4 infants (2.7%) were born with low Apgar score (0-3 score), 2 (1.3%) infants with moderate Apgar score (4-6 score), and 144 (96%) with high Apgar (7-10 score). There was no low Apgar score among participants in the control group and only one infant was born with moderate Apgar score (0.3%) and the others were born (99.7%) with high Apgar score. There was a statistically significant difference between two groups in terms of the Apgar score. Overall, 24 infants (16%) were hospitalized in NICU in case group and 8 infants (2.7%) were hospitalized in NICU in control group.

In terms of neonatal weight in case group, 100 infants were born with normal weight (66.7%), 49 infants with moderate weight (32.7%), and one infant was born with low weight (0.7%). However; in the control group 265 infants were born with normal weight (88.3%), 34 infants with moderate weight (11.3%) and one infant was born with low weight (0.3%). There was a statistically significant difference between two groups in terms of neonatal weight.

In terms of mortality, 4 stillbirths occurred among subjects of the study group, whereas there was no stillbirth in the participants of control group. Results of a logistic regression analysis showed that among the effective factors, neonatal weight was just equally predictive in both groups. Table 1 depicts the distribution of fetal complications among both groups. There were statistically significant differences between two groups for Bradycardia, first minute Apgar score, delivery type and mortality rate. Table 2 shows the frequency distribution for types of delivery between the study and the control groups. Cesarean section occurred in 42.7% of participants in study group and in 17% of subjects in control group. In general, Cesarean section was performed in 25.6% of all participants in both groups.

**Table 1:** Frequency distribution of fetal complications between two groups

Complications	Study group	Control group	*P* value
Meconium	17 (11.3%)	29 (9.7%)	*p*= 0.62
Descent arrest	4 (2.7%)	14 (4.7%)	*p* = 0.44
Bradycardia	42 (28%)	10 (3.3%)	*p* < 0.001
First minute Apgar score (4-6 score)	2 (1.3%)	1 (0.3%)	*p* < 0.001
Mortality	4 (2.7%)	0 (0%)	*p*< 0.001

**Table 2:** Frequency distribution for delivery types between the study and control groups

Groups	Types ofdelivery		

	NVD	C/S	Total
Study	86 (57.3%)	64 (42.7%)	150
Control	249 (83.0%)	51 (17.0%)	300
Total	335 (74.4%)	115 (25.6%)	450

## 4. Discussion

Healthcare providers apply screening strategies to diagnose high-risk situations in order to perform appropriate interventions and obtain better outcomes. Although several techniques are used to monitor fetal well-being in both low and high risk pregnancies such as NST, biophysical profile, etc., the most suitable time to apply these techniques and their diagnostic value to detect fetal complications is controversial. Annually, 3.2 million stillbirths occur. Early detection and timely management of maternal and fetal complications during pregnancy and labor can reduce the rate of stillbirth and prevent maternal and fetal morbidity and mortality. During labor, late detection of maternal and fetal complications such as placental dysfunction and its related hypoxia or poor maternal and fetal tolerance of labor results in stillbirth, neonatal physical and developmental disabilities, and maternal and neonatal mortality and morbidity (Haws, Yakoob, Soomro, Menezes, Darmstadt, & Bhutta, 2009).

Non stress test is one of the available non invasive screening techniques suggested for use in high risk pregnancies during the prenatal period. In addition, NST is used as a screening tool for monitoring fetal well-being via using the relationship between fetal movement and heart rate. Labor is the last stage of pregnancy and it is important to assess feto-placental and utero placental efficient performance.

IN addition, NST is beneficial during the antenatal period, but it is not routinely used during labor or intrapartum phases. During uterine contractions, the blood and oxygen flow decreases temporarily. Thus it is important to assess fetal tolerance of this decrease. The reduction or loss of fetal movements is a warning sign for mothers especially, when it is due to utero placental insufficiency. Several studies have been carried out in order to evaluate maternal and neonatal outcomes related to fetal movement decrease (Kerner, Yogev, Belkin, Ben-Haroush, Zeevi, & Hod, 2004; Malcus, 2004; Liston, Sawchuck, & Young, 2007). Non stress test (NST) is one of the fundamental components of prenatal care. Studies have shown mothers with decreased fetal movements (DFM) are at higher risk of stillbirth, fetal distress, preterm birth, and other related outcomes. Daily fetal movement counting in the 9th month of pregnancy decreased perinatal mortality (Gurneesh & Ellora, 2009). Similarly, in a study by Sidha and Singh (2008), in the absence of any other risk factors interfering in early delivery, daily fetal movement counting was found to be useful in diagnosing at- risk fetuses in low risk pregnancies (Sing & Sidhumk, 2008). This study showed that NST is a useful technique in recognizing high risk fetuses during the latent phase of delivery. Although previous studies have shown the importance of performing NST during prenatal period, the importance of NST during latent phase of labor has been neglected. Since there is a high relationship between DFM and the fetal outcomes, proper and timely management should be planned for any conditions associated with DFM (Frøen, Tveit, Saastad, Børdahl, Stray-Pedersen, Heazell, Flenady, Ruth, & Fretts, 2008). It has been shown that improved DFM management along with uniform information to women decrease stillbirths (Tveit, Saastad, Stray-Pedersen, Børdahl, Flenady, Fretts, & Frøen, 2009). Several case control studies using daily count chart for fetal movements have shown significant differences between that first minute Apgar score below 7, and mortality rate between case and control groups (Shanmugavel, Sodhi, Sandhu, Sidhu, Singh, Katariya, & Khandelwal, 2008). Similarly, in our study, significant differences were found between two groups in terms of morality rate, and the first minute Apgar score below 7. In addition, this study found a significant statistical differences for bradycardia, and delivery type between study and control groups (*p*<0.001). According to results of this study, NST has a high diagnostic value in diagnosis of fetal distress. In order to have an optimal management of labor, NST should be applied during latent phase of labor.

## 5. Conclusion

Results of this study revealed that participants in study group with non reactive NST results had more fetal complications than those with reactive NST results in control group. Since results of current study revealed significant differences between two groups in terms of the delivery type, descent arrest, bradycardia, first minute Apgar score below 7, and mortality rate, this study suggests that NST as a valuable screening technique to be used routinely as a diagnostic test during latent phase of labor. Further research is recommended to explore the relationship between contraction stress test (CST) and fetal and maternal outcomes during the latent phase of delivery. In addition, overall cesarean section rate was high and performed in 25.6% of all participants.
